# Genetic Code Mutations: The Breaking of a Three Billion Year Invariance

**DOI:** 10.1371/journal.pone.0012206

**Published:** 2010-08-20

**Authors:** Wai-Kin Mat, Hong Xue, J. Tze-Fei Wong

**Affiliations:** Applied Genomics Center, Fok Ying Tung Graduate School and Department of Biochemistry, Hong Kong University of Science and Technology, Hong Kong, China; St. Petersburg Pasteur Institute, Russian Federation

## Abstract

The genetic code has been unchanging for some three billion years in its canonical ensemble of encoded amino acids, as indicated by the universal adoption of this ensemble by all known organisms. Code mutations beginning with the encoding of 4-fluoro-Trp by *Bacillus subtilis*, initially replacing and eventually displacing Trp from the ensemble, first revealed the intrinsic mutability of the code. This has since been confirmed by a spectrum of other experimental code alterations in both prokaryotes and eukaryotes. To shed light on the experimental conversion of a rigidly invariant code to a mutating code, the present study examined code mutations determining the propagation of *Bacillus subtilis* on Trp and 4-, 5- and 6-fluoro-tryptophans. The results obtained with the mutants with respect to cross-inhibitions between the different indole amino acids, and the growth effects of individual nutrient withdrawals rendering essential their biosynthetic pathways, suggested that oligogenic barriers comprising sensitive proteins which malfunction with amino acid analogues provide effective mechanisms for preserving the invariance of the code through immemorial time, and mutations of these barriers open up the code to continuous change.

## Introduction

The genetic code selects 23 amino acids for encoding as canonical building blocks for proteins, with twenty standard ones each receiving uniquely assigned codons applicable throughout the entire proteome, and the three additional ones Pyl, Sec and fMet encoded only by shared codons for specific sites in the proteome. Although the universality of this canonical amino acid ensemble among living organisms indicates that the ensemble has not undergone any significant alteration over some three billion years, experimental mutations of the genetic code in *Bacillus subtilis* not only to encode the normally inhibitory analogue 4-fluorotryptophan (4FTrp) as an amino acid building block that supports indefinite cell propagation, but also to reject Trp itself as such a building block, made evident the intrinsic mutability of the genetic code [1]–[3]. Subsequently, encoding of 4FTrp has also been achieved with *E. coli* [4]–[7], and encodings of 5FTrp and 6FTrp with *B. subtilis* [8], [9]. Moreover, proteome-wide encodings have been accomplished for non-indole unnatural amino acids [10]–[13], and encodings of a wide range of unnatural amino acids for specified sites in prokaryotic and eukaryotic proteomes [14]–[19]. These diverse genetic code mutations necessarily raise the question of the nature of mechanisms that have preserved an inherently mutable ensemble of encoded amino acids in an unaltered form throughout the ages. In the present study these mechanisms were investigated by examining the factors determining the success or failure of wildtype and mutant *B. subtilis* cells to propagate on Trp, and 4-, 5- and 6-fluorotryptophans. The findings provided evidence for the important role of oligogenic barriers, consisting of genes for unnatural amino acid-sensitive proteins, in resisting mutational change and safeguarding the timeless stability of the genetic code.

## Results

### Encodings of Fluorotryptophans

**Figure 1:**
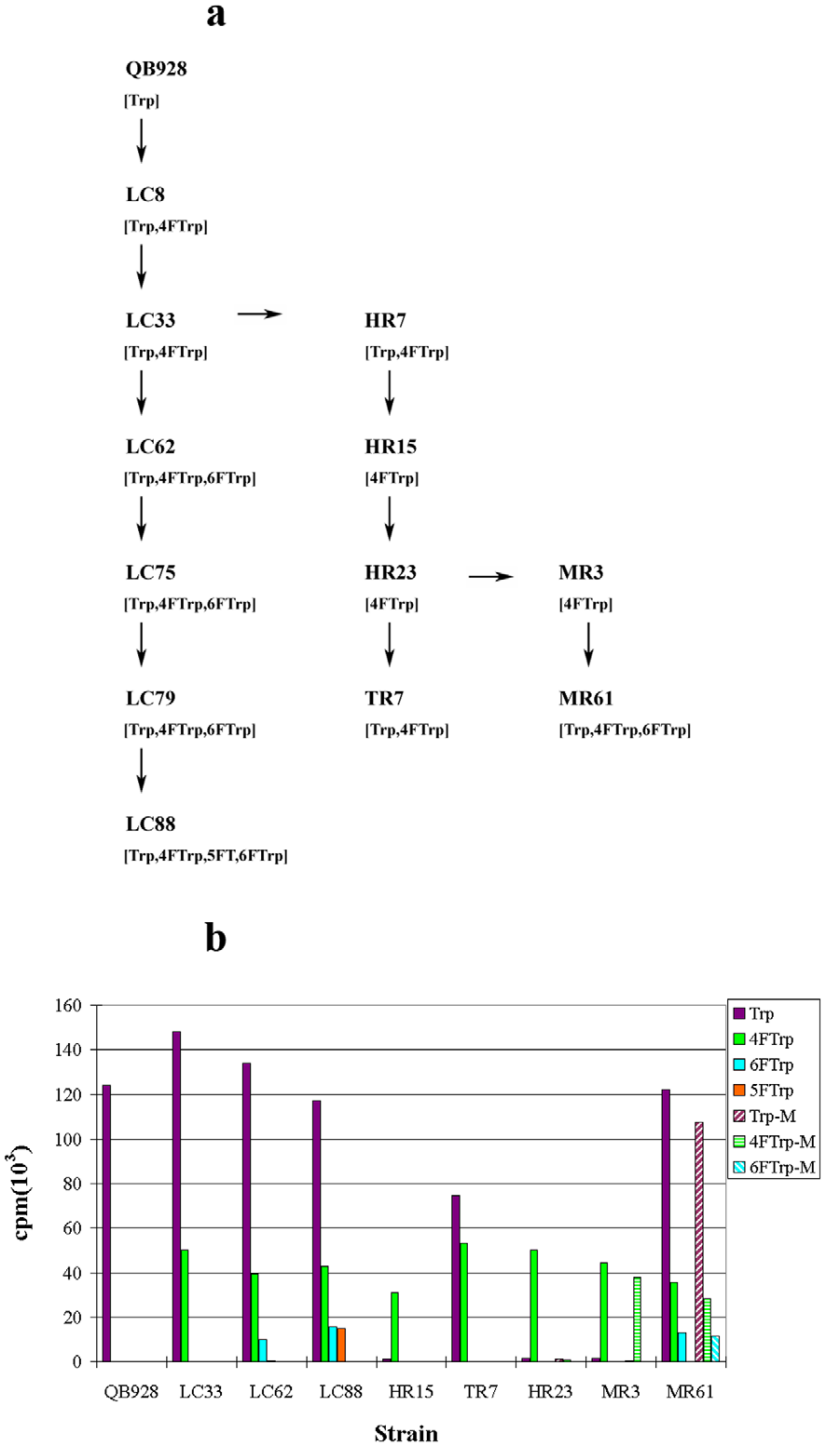
Pathways of genetic code mutations. (a) The successive mutation steps generating various mutant strains starting from the QB928 wildtype are represented by arrows, with the indole amino acid(s) that supported the propagation of each strain shown in brackets below the name of the strain. (b) Growth of various strains on different indole amino acids on agar. For each strain, columns 1–4 from the left represent the amount of growth, measured by ^33^P-phosphate incorporation, on Trp, 4FTrp, 6FTrp and 5FTrp respectively. For the HR23, MR3 and MR61 strains, the additional columns 5–7 represent growth on Trp-minus Met (or Trp-M, i.e., with Met withdrawn from a medium G supplemented by Trp), 4FTrp-minus Met, and 6FTrp-minus Met respectively.

The Trp-auxotroph *Bacillus subtilis* QB928 could propagate on Trp but not its fluoro-analogues 4FTrp, 5FTrp and 6FTrp, but it gave rise after two rounds of mutations to the LC33 strain which readily propagated on both Trp and 4FTrp. These mutations thus expanded the code to enable the proteome-wide encoding of 4FTrp as a propagation-supporting amino acid, replacing Trp at all the 12,625 Trp positions in the *B. subtilis* proteome. As shown in Figure 1a, further mutation of LC33 led to LC62 which could propagate on 6FTrp in addition to Trp and 4FTrp. LC62 had to be mutated to LC75 and LC79, which grew well on 5FTrp in the presence of a decreased concentration of Trp, before LC88 was obtained which propagated on any one of Trp, 4FTrp, 5FTrp and 6FTrp as the sole indole amino acid [8], [9].

**Figure 2:**
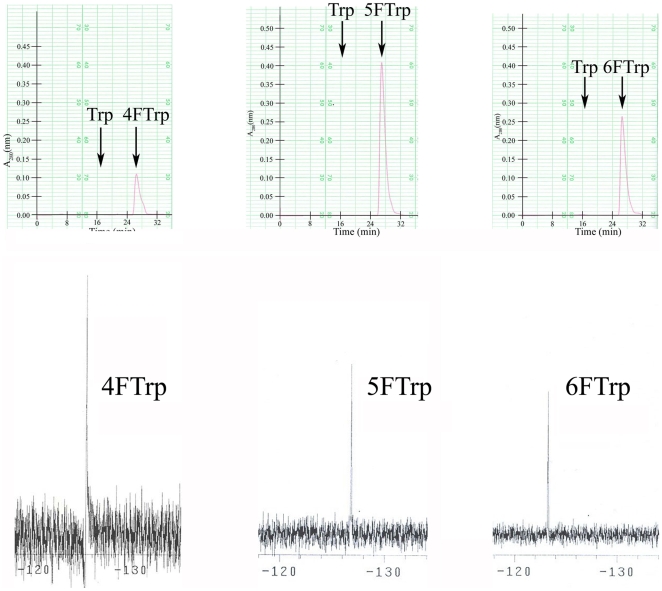
Indole amino acid content of proteins of LC88 cells grown on fluoroTrp. The three two-row vertical panels pertain to protein hydrolysates from cells grown, from the left, on 4FTrp, 5FTrp and 6FTrp. Top row shows that each hydrolysate contained only a fluoroTrp peak but no detectible Trp peak. Bottom row shows the identification of the fluoroTrp peaks from the left as 4FTrp (−125.41 ppm), 5FTrp (−126.84 ppm) and 6FTrp (−123.28 ppm) respectively based on ^19^F NMR.

Whereas the mutation of QB928 to successively LC33, LC62 and LC88 brought about the progressive expansion of the genetic code to allow cell propagation on the unnatural fluoro-analogues, the mutation of LC33 to HR15 acted in the opposite direction and brought about code restriction by expelling long serving Trp from the canonical ensemble of genetically encoded, propagation-supporting amino acids (Figure 1a). Figure 1b shows that the Trp-expulsion was inherited when HR15 was mutated to HR23, a faster growing variant on 4FTrp. In fact, since both HR15 and HR23 failed to propagate on either 6FTrp or 5FTrp, for these strains 4FTrp was the only propagation–supporting indole amino acid. Furthermore, the growth of HR15 and HR23 on 4FTrp but not on Trp clearly indicated that 4FTrp was utilized directly by these strains rather than indirectly via metabolic conversion to Trp. Even in LC88, which grew on Trp, 4FTrp, 5FTrp or 6FTrp, there was no significant conversion of the three fluoro-analogues to Trp prior to incorporation into proteins; protein hydrolysates of LC88 grown on 4FTrp, 5FTrp or 6FTrp contained in each instance only the fluoro-analogue without detectible Trp (Figure 2), in accord with the absence of Trp in proteins from HR15 cells grown on 4FTrp [1].

Altogether, these findings established that the genetic code to-day remains fully capable of expanding and turning over its canonical amino acid alphabet, admitting or expelling amino acids from the alphabet in response to selection.

### Cross Inhibitions

**Figure 3:**
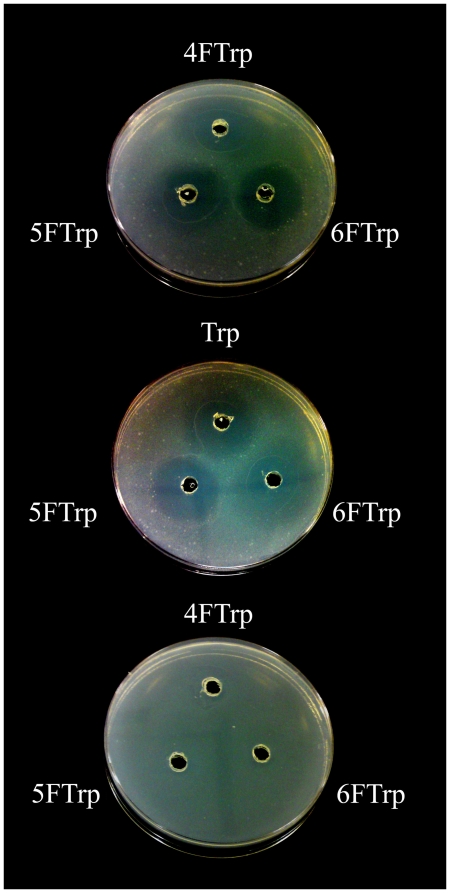
Cross inhibitions between different indole amino acids. Top: QB928 growing on Trp displayed inhibition rings around wells containing 4FTrp, 5FTrp or 6FTrp. Center: HR23 growing on 4FTrp displayed inhibition rings around wells containing Trp, 5FTrp or 6FTrp. Bottom: absence of inhibition rings indicated lack of extensive inhibition by 4FTrp, 5FTrp or 6FTrp against LC88 propagating on Trp.

Trp and its three fuoro-analogues each supported the propagation of some of the *B. subtilis* strains in Figure 1 and failed to do so with other strains. Possible mechanisms for the failures include: the compound is not incorporated into cellular proteins, its incorporation causes one or more of the proteins in the cells to malfunction, or it interferes with some metabolic steps in the cells. Figure 3 shows that the growth of QB928 on Trp was inhibited by 4FTrp, 5FTrp and 6FTrp, each giving rise to an inhibition ring surrounding a well containing the analogue. Similarly, the growth of HR23 on 4FTrp was inhibited by Trp, 5FTrp and 6FTrp; in this instance, colonies growing inside the Trp inhibition zone could be recovered as revertants, e.g. TR7, which regained the ability to grow on Trp (Figure 1b). In contrast, since LC88 could propagate on all four indole compounds, LC88 growing on Trp did not display any inhibition ring due to inhibition by 4FTrp, 5FTrp or 6FTrp. A plausible explanation for this parallelism between non-support of propagation and inhibitory action by the same Trp-analogue is that its failure to support cell propagation might be the consequence of its proteome-wide incorporation into cellular proteins leading to malfunction in one or more essential proteins. Such *incorporation-induced malfunction*, resulting from a misfit between the incorporated residue and the remainder of the protein molecule, would bring about inhibition by the analogue against cell growth on other indole amino acid substrates as well. Such malfunction is therefore entirely in agreement with the observed parallelism, although other factors such as reduced analogue incorporation or interference of metabolic steps by the analogue might also contribute to the propagation failures and cross inhibitions of growth.

### Nutrient Withdrawal Test

In view of the possible role played by incorporation-induced malfunction in determining the ability of *B. subtilis* cells to grow on a fluoroTrp, the finding that just a few rounds of mutations succeeded in allowing *B. subtilis* to propagate on the potent erstwhile inhibitors 4FTrp, 5FTrp or 6FTrp (Figure 1) suggests that the majority of *B. subtilis* proteins were largely tolerant toward the replacement of their Trp residues by 4FTrp, 5FTrp or 6FTrp. It follows that there were in all likelihood only a limited number of analogue**-**sensitive essential proteins encoded by the *B. subtilis* genome that contained critical Trp residue(s) where insertion of fluoroTrp in place of Trp gave rise to gross malfunction. This small collection of analogue-sensitive proteins in effect constituted an oligogenic barrier blocking the propagation of *B. subtilis* on a fluoroTrp. On this basis, mutation of QB928 to LC33 for example could stem from the desensitization of 4FTrp-sensitive proteins so that they remained functional when incorporating either Trp or 4FTrp. Similarly, when LC33 mutated to HR15 and HR23, one or more protein(s) that functioned well with either Trp or 4FTrp would become altered, so that they now functioned adequately with 4FTrp but no longer with Trp [2].

Since all the *B. subtilis* strains in Figure 1 were grown on Trp or a fluoroTrp in medium G which contained a wide range of added nutrients, the postulate that only a small number of genes forming an oligogenic barrier acted against the replacement of Trp by a fluoroTrp can be subjected to verification by the *nutrient withdrawal test*. In this test, the added nutrients were withdrawn one at a time from medium G. The effects of individual withdrawals on LC88 growing on 4FTrp, 5FTrp or 6FTrp were separated into Classes I-IV depending on the percentile growth rate under withdrawal relative to growth on complete medium G (Table 1). Withdrawal of Pro, for instance, produced little reduction of growth rate on 4FTrp, 5FTrp or 6FTrp, and fell into Class I in all three instances. Pro biosynthesis in *B. subtilis* requires γ-Glu-kinase which contains 2 Trp residues, γ-Glu-phosphate reductase with 2 Trp residues and pyrroline-5-carboxylate reductase with one Trp residue. Since these enzymes were not needed in medium G but became essential to growth under Pro withdrawal, the Class I Pro withdrawal effects showed that they all performed adequately when their Trp-positions were filled by 4FTrp, 5FTrp or 6FTrp. Likewise, the numerous enzymes/proteins involved in the biosynthesis or activity of other Class I-II nutrients must have all functioned normally or close to normally when their Trp residues were replaced by a fluoroTrp. In contrast, for each fluoroTrp only 2–4 out of the 31 withdrawals produced a severely inhibitory Class III-IV effect on cell growth. These results indicated that different protein functions in the various nutrient biosynthetic pathways responded unequally to fluoroTrp insertion, and only a minority of the proteins included in the test displayed a severe loss of proper function, in accord with the oligogenic barrier postulate. Therefore, although other factors might also impact on the outcomes of individual nutrient withdrawals, e.g. feedback and other controls acting on the pathways, and interactions between the pathways in the face of changes in metabolic fluxes occasioned by the nutrient withdrawal, the results in Table 1 confirmed the existence of the oligogenic barrier of sensitive proteins as an underlying factor of the surprising finding that gross changes in *B. subtilis* propagations on Trp and fluoroTrps could be brought about by just several rounds of mutations (Figure 1).

**Table 1:** Application of nutrient withdrawal test to LC88 cells growing on 4FTrp, 5FTrp or 6FTrp.

	Growth on 4FTrp	Growth on 5FTrp	Growth on 6FTrp
Class I: 80%–100% growth rate	A,T,C,G,U, Ala, Arg, Asn, Asp, Cys, Gly, Gln, Glu, His, Ile, Lys, Pro, Ser, Val, p-aminobenzoic acid, biotin, folic acid, niacinamide, pantothenate, pyridoxal, ribitol, riboflavin, thiamine.	T,C,G,U, Ala, Arg, Asn, Cys, Gly, Gln, Glu, His, Ile, Lys, Pro, Ser, Thr,Val, p-aminobenzoic acid, biotin, folic acid, niacinamide, pantothenate, pyridoxal, ribitol, riboflavin, thiamine	T,C,G,U, Ala, Arg, Asn, Asp, Cys, Gly, Gln, Ile, Lys, Pro, Ser, Val, p-aminobenzoic acid, biotin, folic acid, niacinamide, pantothenate, pyridoxal, ribitol, riboflavin, thiamine
Class II: 40%–79% growth rate	Thr	A, Asp	A, His
Class III: 10%–39% growth rate	Leu	Leu	Leu, Thr
Class IV: 0%–9% growth rate	Met	Met	Glu, Met

The various nutrients withdrawn one at a time from medium G are shown in columns 2–4, where the growth effect due to each withdrawal is grouped into Classes I-IV depending on the growth rate observed under the withdrawal as a percentage of the control growth rate in complete medium G.

Mutations beyond HR23 in Figure 1 yielded further demonstrations of code mutability. Met was a Class IV nutrient for not only LC88 (Table 1), but also HR23, growing on 4FTrp (Figure 1b). However, when HR23 was mutated to MR3, the Met-related barrier to cell propagation on 4FTrp was reduced, and the MR3 cells succeeded to propagate on 4FTrp even in the absence of Met. When MR3 was further mutated to MR61, the latter inherited the ability to propagate on 4FTrp in the absence of added Met, and also gained the capacity to propagate on Trp and 6FTrp. Thus the success or failure of *B. subtilis* to propagate on Trp or one of the fluoroTrps was subject to continuous alterations as sensitive proteins comprising the particular oligogenic barrier in each instance were mutated.

## Discussion

The shuffling of Trp and its fluoro-analogues in and out of the encoded, propagation-supporting amino acid alphabet established that the genetic code is open to continuous mutational change. This intrinsic code mutability with respect to the amino acid alphabet provides strong support for the occurrence of active early code expansions at the dawn of life as the code coevolved with amino acid biosynthetic pathways that brought new amino acid building blocks to proteins to enhance their functions [20]–[24]. Therefore the chemical versatility of the amino acid alphabet underwritten by the universal genetic code was not the outcome of fortuitous happenstance but the result of nature's relentless search for excellence in protein performance. The timeless constancy of this alphabet throughout the living world only attests to the remarkable effectiveness of oligogenic barriers acting as conservative elements against any alteration of the code once excellence was achieved in terms of the versatility and balance of the member amino acids recruited into the universal genetic code [21]–[22].

The mutability of the genetic code is by no means confined to *B. subtilis*, and *E. coli* also has been mutated to yield an *unColi* B7-3 strain that propagated on 4FTrp without detectible Trp incorporation into proteins [4]–[7]. In addition to the mandatory growth dependence of HR15 and HR23 on 4FTrp, mandatory growth dependence on the site-specific utilization of an unnatural amino acid has been achieved through phenotypic suppression: an *E. coli* thymidylate synthase (*thy A*) Arg126Leu mutant could grow only in the presence of azaLeu, which restored a positive charge to residue-126 and thereby enzyme function [10]. As well, some aminoacyl-tRNA synthetases (aaRS) were found to display strikingly low reactivities toward tRNAs from other species, especially when the other species came from the opposite side of an aaRS-tRNA reactivity divide separating the Archaea-Eukarya and Bacteria blocs [25]. Based on such deficient cross-bloc reactivities, orthogonal archaeal aaRS-tRNA pairs could be devised and introduced into *E. coli* incurring minimal reactions with host aaRS and tRNAs [14]. This orthogonal aaRS-tRNA approach has brought about the site-specific encodings of a wide range of unnatural amino acids in bacterial and eukaryotic cells [15]–[19].

These proteome-wide and site-specific mutations of the genetic code overturning the rigorously preserved code have opened wide the door to the recruitment of unnatural amino acids into the code. As a result, protein structures can be permutated at not only the sequence level but also the level of the amino acid alphabet. This has made possible a deepening of the understanding of protein structure-function relationships, and protein engineering is no longer limited to the 23-amino acid alphabet of to-day [26], [27]. Thus the known living world, characterized by its uniform, straitjacket amino acid alphabet, has now been transformed into a sequel to life equipped with a freely variable alphabet [28].

Moreover, it was suggested that, because the universal amino acid alphabet has been a constant attribute of all earthly life throughout the ages, *B. subtilis* HR15 represents an entirely new type of life [29]. On this basis, 4FTrp-utilizing *E. coli* B7-3 was named *unColi* B7-3 [4], [5], the various strains in Figure 1a would be *unSubtilis* strains, and the organisms bearing genetic codes with site-specific expansions [14]–[19] would likewise be *unColi*, *unYeast* etc. To describe this emergent profusion of novel life forms, it has been proposed [30] that free living organisms that depart from earthly life with respect to one or more of the universal, defining building blocks or attributes of earthly life may be referred to as *synthetic life* to distinguish them from natural life, synthetic plasmids, synthetic viruses, synthetic parasites, synthetic organelles, computational artificial life, as well as constructs of synthetic biology which carry crafted assemblages of natural and modified genes but nonetheless adhere to such universal attributes of earthly life as the canonical 23-amino acid alphabet of proteins, TCAG alphabet of DNA, UCAG alphabet of RNA, and bilayer cell membranes consisting of ester and ether lipids.

The *unSubtilis*, *unColi* and *unYeast* organisms have signaled the open-ended scope of synthetic life freed from the stringent confines of the universal building blocks and attributes inherited from three billion years of biological evolution. On the other hand, the recent construction of a bacterial cell controlled by a chemically synthesized genome has signaled the open-ended scope of fashioning organismic genomes by design [31]. The convergence of these two momentous developments suggests that future organisms may be constructed combining both novel building blocks and designer genome sequences. As has been emphasized [30], how this bold new world will unfold will be a fascination to behold as much as a road that needs to be charted with great care.

## Materials and Methods

### Mutant Isolation

Different *B. subtilis* strains bearing a mutant genetic code were isolated starting from Trp-auxotroph QB928 (*aroI906 purB33 dal trpC_2_*) in medium G containing a range of added nutrients and supplemented with 5 µg/ml of Trp or one of the fluoroTrps, as in the isolation of LC33 and HR15 [1]. LC62 was isolated from LC33 through selection for the ability to form colonies on 6FTrp; LC75 was obtained from LC62, and LC79 from LC75, through selection for the ability to form colonies on 5FTrp in the presence of a decreased concentration of Trp, eventually giving rise to LC88 which could propagate on 5FTrp in the absence of Trp. HR23 was a faster growing variant of HR15 on 4FTrp, and TR7 was isolated from HR23 under Trp-inhibition as a revertant that regained the ability to grow on Trp [8].

Growth of LC62, LC88, TR7, HR23, MR3, MR61 and strains reported earlier on Trp, 4FTrp, 5FTrp or 6FTrp on agar were measured by ^33^P-phosphate incorporation as described [1].

### Indole Amino Acid Content

Cells grown in suspension in medium G containing 5 µg/ml of one of the fluoroTrps were thoroughly washed. Soluble proteins precipitated at 80% ammonium sulfate were extensively dialyzed, followed by hydrolysis with 2 M mercaptoethanesulfonic acid for 38 hours at 110°C [32]. After acid removal on Waters µBondapak-C18 column, a Waters PicoTag column for hydrolysate amino acids was employed to separate any Trp from the slower running fluoroTrps in the hydrolysate using 0.14 M Na acetate, 3.6 mM triethylamine-acetate at pH 6.4 and 6% acetonitrile as eluent. The fluoroTrp contained in each hydrolysate was identified based on ^19^F NMR in DMSO-d_6_ using a Varian Mercury-300 spectrometer operating at 282.35 MHz with trifluoroacetic acid reference set at −75 ppm.

### Cross Inhibitions between Different Indole Amino Acids

To detect possible inhibition of QB928 [1] growth by 4FTrp, 5FTrp or 6FTrp, QB928 cells were grown overnight in liquid medium G containing 5 µg/ml Trp, and diluted to A_450_ of 0.004. A 5 ml volume of the cell suspension was placed on agar plate made up with medium G containing 5 µg/ml Trp, and then removed. After allowing the agar gel to dry, three holes were punched out in the agar gel, and a 50 µl aliquot of 0.1 M 4FTrp, 5FTrp or 6FTrp in dimethyl sulfoxide was placed into a well. After incubation at 37°C for 24 hours, occurrence of growth inhibition in each instance was detected by the appearance of a cleared inhibition ring surrounding the well. Possible inhibition of LC88 [8] growth on Trp by 4FTrp, 5FTrp or 6FTrp was similarly tested. To detect possible growth inhibition of HR23 [8] cells growing on 4FTrp by Trp, 5FTrp or 6FTrp, the cells grown in liquid medium G containing 5 µg/ml 4FTrp were diluted to only A_450_ of 0.5 before placement on agar plate made up with medium G containing 5 µg/ml 4FTrp, and then removed. A 50 µl aliquot of 0.01 M Trp, 5FTrp or 6FTrp in dimethyl sulfoxide was placed into a well on the gel for observation of any inhibition.

### Nutrient Withdrawal Test

LC88 cells [8] were grown on 4FTrp, 5FTrp or 6FTrp in either complete medium G or medium G-minus from which one of the nutrients in complete medium G was withdrawn. Based on the percentile growth rate in medium G-minus relative to that in medium G, the effect of the withdrawal of each nutrient was grouped into Classes I-IV. The medium G-minus growth rate was 80%–100% of the medium G rate for Class I, 40%–79% for Class II, 10%–39% for Class III, and 0%–9% for Class IV.

## References

[1] Wong JT (1983). Membership mutation of the genetic code: Loss of fitness by tryptophan.. Proc Nat Acad Sci USA.

[2] Wong JT, Ricard J, Cornish-Bowden A (1984). Evolution and mutation of the amino acid code.. Dynamics of Biochemical Systems.

[3] Bronskill PM, Wong JT (1988). Suppression of fluorescence of tryptophan residues in proteins by replacement with 4-fluorotryptophan.. Biochem J.

[4] Bacher JM, Ellington AD (2001). Selection and characterization of Escherichia coli variants capable of growth on an otherwise toxic tryptophan analogue.. J Bacteriol.

[5] Bacher JM, Bull JJ, Ellington AD (2003). Evolution of phage with chemically ambiguous proteomes.. BMC Evol Biol.

[6] Bacher JM, Hughes RA, Wong JT, Ellington AD (2004). Evolving new genetic codes.. Trends Ecol Evol.

[7] Bacher JM, Ellington AD (2007). Global incorporation of unnatural amino acids in Escherichia coli.. Methods Mol Biol.

[8] Mat WK, Xue H, Wong JT (2004). Genetic encoding of 4-, 5-, and 6-fluorotryptophan residues: role of oligogenic barriers.. Am Soc Microbiol 104^th^ Meeting.

[9] Wong JT, Wong JT, Lazcano A (2009). Genetic code.. Prebiotic Evolution and Astrobiology.

[10] Lemeignan B, Sonigo P, Marliere P (1993). Phenotypic suppression by incorporation of an alien amino acid.. J Mol Biol.

[11] Doring V, Mootz HD, Nangle LA, Hendrickson TL, de Crecy-Lagard V (2001). Enlarging the amino acid set of Escherichia coli by infiltration of the valine coding pathway.. Science.

[12] Pezo V, Metzgar D, Hendrickson TL, Waas WF, Hazebrouck S (2004). Artificially ambiguous genetic code confers growth yield advantage.. Proc Natl Acad Sci USA.

[13] Tang Y, Tirrell DA (2002). Attenuation of the editing activity of the Escherichia coli leucyl-tRNA synthetase allows incorporation of novel amino acids into proteins in vivo.. Biochemistry.

[14] Santoro SW, Anderson JC, Lakshman V, Schultz PG (2003). An archaealbacteria-derived glutamyl-tRNA synthetase and tRNA pair for unnatural amino acid mutagenesis of proteins in Escherichia coli.. Nucl Acid Res.

[15] Mehl RA, Anderson JC, Santoro SW, Wang L, Martin AB (2003). Generation of a bacterium with a 21^st^ amino acid genetic code.. J Am Chem Soc.

[16] Sakamoto K, Hayashi A, Sakamoto A, Kiga D, Nakayama H (2002). Site-specific incorporation of an unnatural amino acid into proteins in mammalian cells.. Nucl Acid Res.

[17] Kohrer C, Sullivan EL, RajBhandary UL (2004). Complete set of orthogonal 21^st^ aminoacyl-tRNA synthetase-amber, ochre and opal suppressor tRNA pairs: concomitant suppression of three different termination codons in an mRNA in mammalian cells.. Nucl Acid Res.

[18] Cropp TA, Anderson JC, Chin JW (2007). Reprogramming the amino acid substrate specificity of orthogonal aminoacyl-tRNA synthetases to expand the genetic code of eukaryotic cells.. Nature Protocols.

[19] Lee HS, Guo J, Lemke EA, Dimla RD, Schultz PG (2009). Genetic incorporation of a small environmentally sensitive fluorescent probe into proteins in Saccharomyces cerevisiae.. J Am Chem Soc.

[20] Wong JT (1975). A co-evolution theory of the genetic code.. Proc Natl Acad Sci USA.

[21] Wong JT (1976). The evolution of a universal genetic code.. Proc Natl Acad Sci USA.

[22] Wong JT, Xue H, Palyi G, Zucchi C, Caglioti L (2002). Self-perfecting evolution of heteropolymer building blocks and sequences as the basis of life.. Fundamentals of Life.

[23] Wong JT (2005). Coevolution theory of the genetic code at age thirty.. BioEssays.

[24] Wong JT (2007). Coevolution theory of the genetic code: a proven theory.. Orig Life Evol Biosph.

[25] Kwok Y, Wong JT (1980). Evolutionary relationship between Halobacterium cutirubrum and eukaryotes determined by use of aminoacyl-tRNA synthetases as phylogenetic probes.. Canad J Biochem.

[26] Bock A (2001). Invading the genetic code.. Science.

[27] Budisa N (2006). Engineering the Genetic Code..

[28] Cohen P (2000). Life the sequel.. New Scientist.

[29] Hesman T (2000). Code breakers. Scientists are changing bacteria in a most fundamental way.. Science News.

[30] Wong JT, Xue H, Luisi PL, Chiarabelli CC (2010). Synthetic genetic codes as the basis of synthetic life.. Chemical Synthetic Biology.

[31] Gibson DG, Glass JI, Lartigue C, Noskov VN, Chuang RY (2010). Creation of a bacterial cell controlled by a chemically synthesized genome.. Sciencexpress.

[32] Penke B, Ferenczi R, Kovács K (1974). A new acid hydrolysis method for determining tryptophan in peptides and proteins.. Anal Biochem.

